# The Synergetic Reduction of the Condensation Degree of Dissolved Lignin (DL) during the Refining Process of Wheat Straw Biomass Based on the MA/O_3_ System

**DOI:** 10.3390/molecules29133228

**Published:** 2024-07-08

**Authors:** Xiuguang Chen, Zhulan Liu, Zhenyu Zhou, Renai Li, Lizi Li, Yunfeng Cao

**Affiliations:** 1 Jiangsu Co-Innovation Center for Efficient Processing and Utilization of Forest Resources, Jiangsu Provincial Key Lab Pulp & Paper Science and Technology, Nanjing Forestry University, Nanjing 210037, China; chenxiuguang@njfu.edu.cn (X.C.); lirenai@njfu.edu.cn (R.L.); 2 Zhejiang Kan New Materials Co., Ltd., Lishui 323300, China; cxg19980906@163.com (Z.Z.); 18168034156@163.com (L.L.)

**Keywords:** wheat straw, MA, O_3_, component separation, lignin

## Abstract

Lignin, a natural pol2ymer with a complex structure that is difficult to separate, is prone to C-C bond condensation during the separation process. To reduce the condensation of lignin, here, a novel method is proposed for separating the components by using a combination of maleic acid (MA)/ozone (O_3_) to co-treat wheat straw. The removal of lignin, glucan, and xylan was 38.07 ± 0.2%, 31.44 ± 0.1%, and 71.98 ± 0.1%, respectively, under the conditions of ball-milling of wheat straw for 6 h, reaction temperature of 60 °C, and O_3_ holding time of 9 min. Lignin-rich solutions were collected to extract the dissolved lignin (DL) after washing the treated samples. The DL obtained under MA/O_3_ conditions had a carboxyl group (-COOH) content of 2.96 mmol/g. The carboxyl group of MA underwent esterification with the hydroxyl group (-OH) at the γ position of lignin and O_3_ reacted on the positions of the lignin side chain or the phenolic ring, resulting in a break in the side chain and the opening of the phenolic ring to introduce the carboxyl group. The 2D-HSQC-NMR results revealed that the phenolic ring-opening reaction of lignin in the presence of O_3_ was essentially free of β-β and β-5 condensation bonds.

## 1. Introduction

Lignocellulosic biomass stands as the most abundant natural resource, stored in various forms such as in agricultural residues [1], wood [2], graminoids [3], waste paper [4], kitchen waste [5], etc. These materials are recognized as cost-effective feedstocks for producing biomaterials, biofuels, and platform chemicals. Among agricultural wastes, wheat straw is particularly rich in lignin, cellulose, and hemicellulose. Lignin, a natural polymer, boasts a three-dimensional network composed of guaiacyl (G), syringyl (S), and p-hydroxyphenyl (H) units interconnected by ether (C-O-C) and carbon–carbon (C-C) bonds [6]. This complex structure endows lignin with numerous functional groups, such as hydroxyl (-OH), double bonds (C=C), and ether bonds, making it highly suitable for antimicrobial [7] and anti-ultraviolet applications [8]. Additionally, lignin’s excellent biodegradability, reactivity, and biocompatibility make it a promising candidate for use as an antimicrobial agent, drug and gene carrier, and wound healing material [9,10,11,12]. Its potential extends to biomedical applications, including drug delivery and tissue engineering [13,14,15,16]. Some scholars have already elaborated on the biomedical applications of lignin [17].

In biomass systems, lignin reinforces structural components through physical or chemical binding, thereby enhancing the mechanical strength of the cell wall but also hindering the separation of these components [18]. Moreover, the structure of lignin also contains hydroxyl groups and many polar groups, which can form strong intramolecular and intermolecular hydrogen bonds, making lignin extremely stable and difficult to dissolve [19]. The chemical separation process of lignocellulose is usually performed with the help of inorganic acids [20], alkalis [21], ionic liquids [22], or organic solvents in total solvent systems [23]. However, the traditional method has the disadvantages of difficult solvent recovery, low lignin utilization, the high cost of ionic liquids, and the severe C-C bond condensation of lignin under vigorous separation conditions [24]. Condensed lignin in particular has increased molecular weight and decreased reactivity, rendering it unfavorable for further depolymerization into platform molecular compounds [25] and the production of high-value products.

Compared with conventional methods, the use of solid organic acid such as maleic acid (MA) is versatile, and both wood feedstocks [26] and herbaceous feedstocks [27] can be rapidly delignified at atmospheric pressure. Cai et al. found that 49.4% of lignin was removed from birch at 100 °C, with a solid–liquid ratio of 1:10, reaction time of 60 min, and acid concentration of 50 wt% [28]. Su et al. found that 71% of lignin was removed from wheat straw at 120 °C, with a solid–liquid ratio of 1:15, reaction time of 90 min, and acid concentration of 60 wt% [29]. They also revealed that the treatment using MA hydrotropic fractionation also introduced carboxyl groups into lignin and enhanced antioxidant activity. However, the lignin obtained at high temperatures (>100 °C) with high solid–liquid ratios (>1:10) and acid concentrations (>50 wt.%) had a severe degree of condensation, especially the S and G units. Although the degree of lignin condensation was reduced compared to traditional methods, condensed lignin is still a problem that cannot be ignored. Reducing the reaction temperature and the solid–liquid ratio has limited effects on reducing the degree of lignin condensation and decreases the removal ratios of the components of wheat straw. To further reduce lignin condensation, this study introduced O_3_, which has great oxidizing properties.

O_3_ is an allotrope of oxygen, a colorless gas at room temperature and pressure, and soluble in water. Among the common oxygen-containing reactive groups, O_3_ is the strongest oxidant other than hydroxyl radicals, with an oxidation potential of 2.07 V. It has been reported that O_3_ can attack the C_3_-C_4_ position of lignin to form a muconic acid structure [30], which introduces carboxyl groups into the lignin structure. In addition, the lignin side chain can be oxidized with O_3_, destroying the double bond structure while introducing hydroxyl and carboxyl groups to increase lignin reactivity [31,32]. It is worth noting that O_3_ is extremely unstable [33]; the higher the temperature, the shorter the half-life, and it is not easy to transport or store, which determines that it must be prepared on-site. Therefore, the present study envisaged the introduction of O_3_ into an MA system for rapid lignin removal at lower treatment temperatures and reduced lignin condensation.

**Figure 1:**
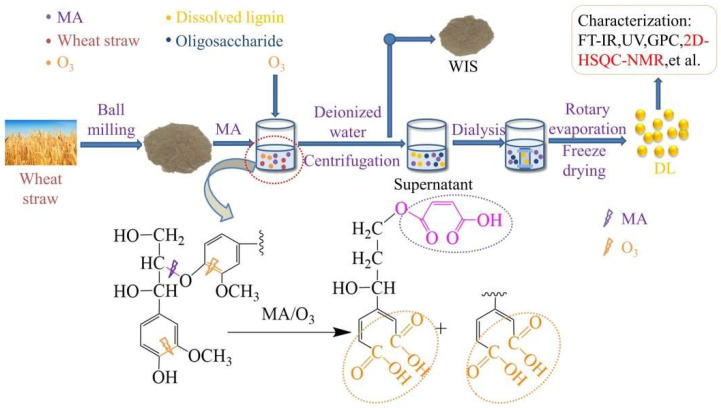
Experimental flow chart and degradation mechanism of lignin.

In this study, MA and O_3_ were combined to separate wheat straw fractions and investigate the effects of wheat straw pulverization degree, O_3_ holding time, and reaction temperature on the removal ratio of the fractions. The experimental procedure and reaction mechanism are depicted in Figure 1. Optimal conditions yielded removal ratios of 38.07 ± 0.2% for lignin, 31.44 ± 0.1% for glucan, and 71.98 ± 0.1% for xylan. Remarkably, the obtained lignin was free from β-β and β-5 condensation bonds and exhibited a carboxyl group content of 2.96 mmol/g.

## 2. Results and Discussion

### 2.1. Effect of MA/O_3_ on the Removal of Components of Wheat Straw

Table S1 shows the component removal ratio of the wheat straw after treatment under different conditions. For ground wheat straw without ball-milling, the removal ratios initially increased with time but subsequently decreased under constant temperature conditions. This trend was attributed to the dense structure and high crystallinity of the raw material, which impeded the penetration of acid and O_3_ into the wheat straw [34]. Generally, the removal ratios decreased as the temperature increased to 70 °C or 80 °C when O_3_ was used as the sole treatment. This decline was due to the decreased stability of O_3_ (shorter half-life and accelerated decomposition) at higher temperatures, which reduced its reactivity with the feedstock. The removal ratios of components of wheat straw were higher under MA/O_3_ and O_3_/H_2_SO_4_ conditions compared to O_3_ treatment alone. The presence of acid promoted the reaction between O_3_ and the components of wheat straw [35]. The component removal ratio after MA/O_3_ treatment was higher than that of O_3_/H_2_SO_4_ treatment, indicating that the synergistic effect of MA and O_3_ was superior to that of O_3_ and H_2_SO_4_.

At 60 °C with an O_3_ holding time of 6 min, the lignin removal ratio under MA/O_3_ treatment was 25.21%, an increase of 6.41 percentage points compared to the 18.80% observed with MA treatment alone. When the temperature was raised to 80 °C, the lignin removal ratio increased from 23.75% to 26.70%, representing an increase of only 2.95 percentage points. This demonstrates that although increasing the temperature could enhance the MA reaction, the gain in lignin removal ratio at 80 °C was limited due to O_3_ decomposition. With an O_3_ holding time of 6 min, lignin removal was hindered by the O_3_ decomposition induced by the temperature increase.

The carboxyl group in the MA structure could react with the hydroxyl group in xylan or dextran to form an ester bond, introducing a carboxyl group into the cellulose. However, this reaction did not cause significant removal of dextran or xylan, but more of a structural change. As shown in Table S1, the removal of xylan and dextran was also relatively high with MA/O_3_ treatment because the O_3_ was a non-selective substance that could react with lignin, dextran, and xylan. O_3_ underwent oxidation reactions with carbohydrates, converting reducing end groups to carboxyl groups, converting hydroxyl groups to carbonyl groups, and breaking ligand bonds through ozonolysis [36].

**Figure 2:**
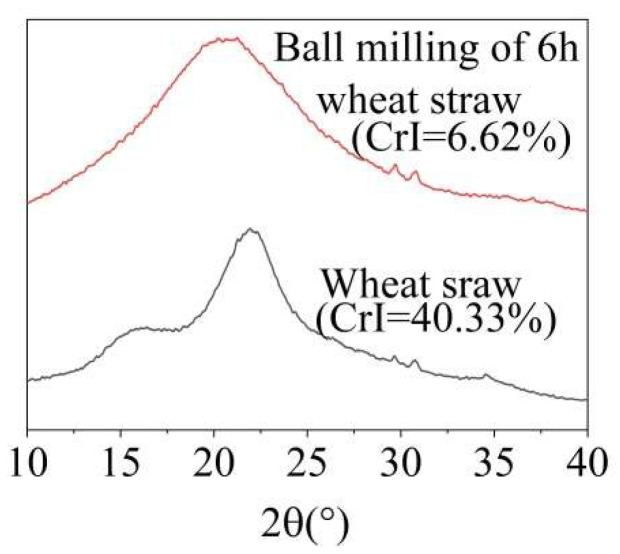
X-ray diffraction patterns of wheat straw before and after ball-milling.

If the wheat straw was ball-milled for 6 h, the rigid structure made of cellulose, hemicellulose, and lignin was destroyed and the crystallinity was reduced (Figure 2). The ball-milled wheat straw was more accessible to MA and O_3_, allowing these agents to penetrate the straw more thoroughly and facilitating the removal of lignin. As shown in Table S1, the overall lignin removal ratio from ball-milled wheat straw increased significantly. As shown in Table 1, under the optimal conditions of 60 °C and an O_3_ holding time of 9 min, the lignin removal ratio reached 38.07%, which was 11.37% higher than the 23.32% achieved with ground wheat straw without ball-milling. However, as the temperature increased, the lignin removal ratio slightly decreased, likely due to the accelerated, ineffective decomposition of O_3_ at higher temperatures.

The oxidation of hydroxylated (HO•) and hydroperoxyl (HOO•) radicals produced by O_3_ decomposition affected the removal of glucan and xylan. HOO• free radicals oxidize the reducing terminal groups of carbohydrates to carboxyl groups (-COOH); HO• free radicals oxidized both the reducing terminal groups and the aliphatic hydroxyl groups to carboxyl groups and formed the ketol structure on the polysaccharide chain, leading to chain breakage [37].

After treatment under the conditions of 6 h of ball-milling, reaction temperature of 60 °C, and O_3_ holding time of 9 min, the removal ratios of lignin, xylan, and dextran reached 38.07%, 71.98%, and 31.44%, respectively.

### 2.2. Particle Size, Zeta Potential, and Carboxyl Content of Lignin

**Figure 3:**
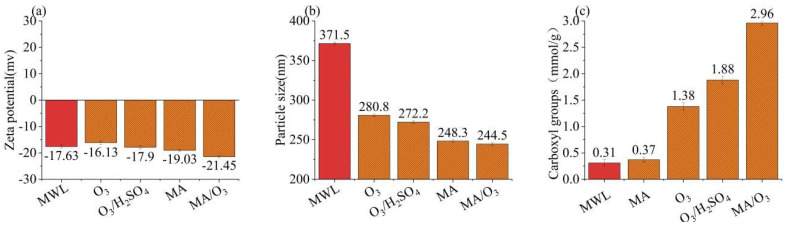
Zeta potential (**a**), particle size (**b**), and carboxyl groups (**c**) of MWL and DL (reaction conditions: ball-milling time: 6 h, T: 60 °C, O_3_ holding time: 9 min).

In Figure 3a,b, the zeta potential and particle size of the DL under the MA/O_3_ condition were −21.45 mV and 221.6 nm, which was smaller than that of MWL (371.5 nm). This indicated that the lignin underwent significant fragmentation due to the action of O_3_ and MA. In Figure 3c, the carboxyl group content of DL obtained under the MA/O_3_ conditions was 2.96 mmol/g, whereas the carboxyl group contents of MWL and DL obtained from MA treatment alone were just 0.31 mmol/g and 0.37 mmol/g, respectively. This suggested that a substantial number of carboxyl groups were introduced through the esterification by MA [28] and the oxidation by O_3_ [32,38]. Due to the introduction of a large number of hydrophilic carboxyl groups, the lignin from the MA/O_3_ system needed to be dissolved in the more polar deuterated water (D_2_O) in the subsequent 2D-HSQC-NMR experiments, while the lignin obtained from the MA system could only be dissolved in the less polar dimethyl sulfoxide-d_6_ (DMSO-d_6_).

### 2.3. UV and FT-IR Spectra Analysis

**Figure 4:**
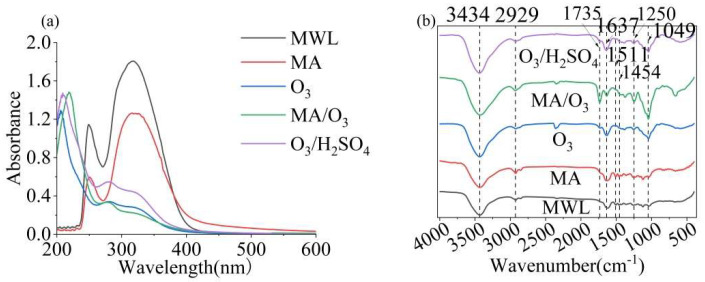
UV (**a**) and FT-IR (**b**) of MWL and DL (reaction conditions: ball–milling time: 6 h, T: 60 °C, O_3_ holding time: 9 min).

In Figure 4a, three absorbance bands can be observed at approximately 219 nm, 284 nm, and 324 nm. The introduction of two methoxy groups into the phenolic ring resulted in a red shift of the maximum absorption of lignin from 280 to 284 nm. This indicated that DL contained a lower amount of S units than the previously extracted lignin [39].

The FT-IR spectrum of lignin, as shown in Figure 4b, exhibited characteristic absorption bands at 3434 cm^−1^ and 2929 cm^−1^, which were attributed to -OH and -CH groups, respectively. The strong C=O absorption band at 1735 cm^−1^ was indicative of the esterification of MA and the potent oxidative properties of O_3_, which introduced carboxyl groups into the lignin structure [40]. Additionally, the -OH and C=O signals of DL obtained under MA/O_3_ conditions were significantly stronger compared to those obtained under other conditions. The characteristic absorption bands at 1637 cm^−1^, 1511 cm^−1^, and 1454 cm^−1^ corresponded to the vibrational modes of the phenolic ring backbone [41]. The vibrational absorption at 1250 cm^−1^ in the lignin was attributed to the C-O bond stretching vibration of the guaiacyl (G) [42]. The presence of hemicellulose in the lignin was suggested by the characteristic absorption at 1049 cm^−1^.

### 2.4. Thermal Stability Analysis

**Figure 5:**
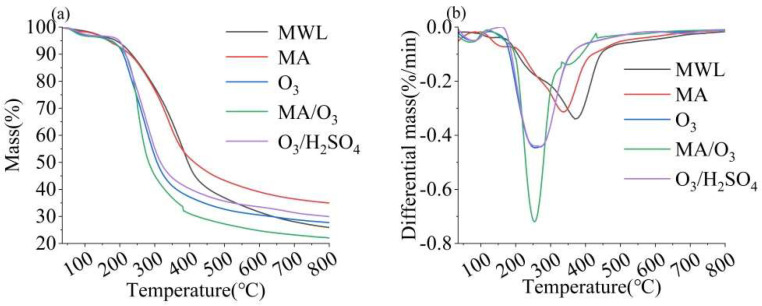
TG (**a**) and DTG (**b**) of MWL and DL (reaction conditions: ball–milling time: 6 h, T: 60 °C, O_3_ holding time: 9 min).

The TG and DTG analyses of lignin are shown in Figure 5, from which the maximum pyrolysis temperature (T_m_) and amount of residual carbon of the lignin for each condition were derived, presented in Table S2. The T_m_ of DL obtained from the MA/O_3_ condition was 256 °C and the residual carbon was 22.02%. Both the T_m_ and the residual carbon contents were significantly lower than those observed for lignin treated with O_3_, MA, or O_3_/H_2_SO_4_. The Tm of lignin was influenced by the content of β-O-4 bonds and the M_w_, which were the two most critical factors [43]. The reduction in T_m_ indicated that the thermal stability of lignin was reduced after MA and O_3_ treatments. This reduction was attributed to the depolymerization reaction induced by MA and the oxidation reaction of O_3_, which broke the β-O-4 bonds in the lignin, leading to a decrease in its molecular weight [29,31,44].

### 2.5. Molecular Weight Analysis

Table 2 shows the M_W_, M_n_, and PI for each of the lignins. Compared with MWL, the M_W_ and M_n_ of DL obtained under MA/O_3_ conditions were significantly smaller, and the polydispersity index of the samples was on the large side, reaching 3.97. This was attributed to the strong oxidizing properties of O_3_ [45] and its significantly accelerated molecular motion rate at 60 °C compared to ambient temperature. O_3_ continuously penetrated the wheat straw and reacted with lignin, leading to the opening of the phenolic ring and oxidation of the side chains [46]. The reactions between O_3_ and the lignin structures are illustrated in Figure S1. Due to the inhomogeneity of lignin distribution in the wheat straw [47], the reaction degree of lignin with MA or O_3_ varied, resulting in polydisperse M_w_.

The Mw of DL under O_3_/H_2_SO_4_ conditions was smaller than that obtained with the O_3_ treatment alone. However, the Mw of DL under MA/O_3_ conditions was even lower than that under O_3_/H_2_SO_4_ conditions. This indicated that the combination of MA and O_3_ produced synergistic effects on the lignin, making the lignin macromolecules more susceptible to fragmentation.

### 2.6. 2D-HSQC-NMR Spectra Analysis

**Figure 6:**
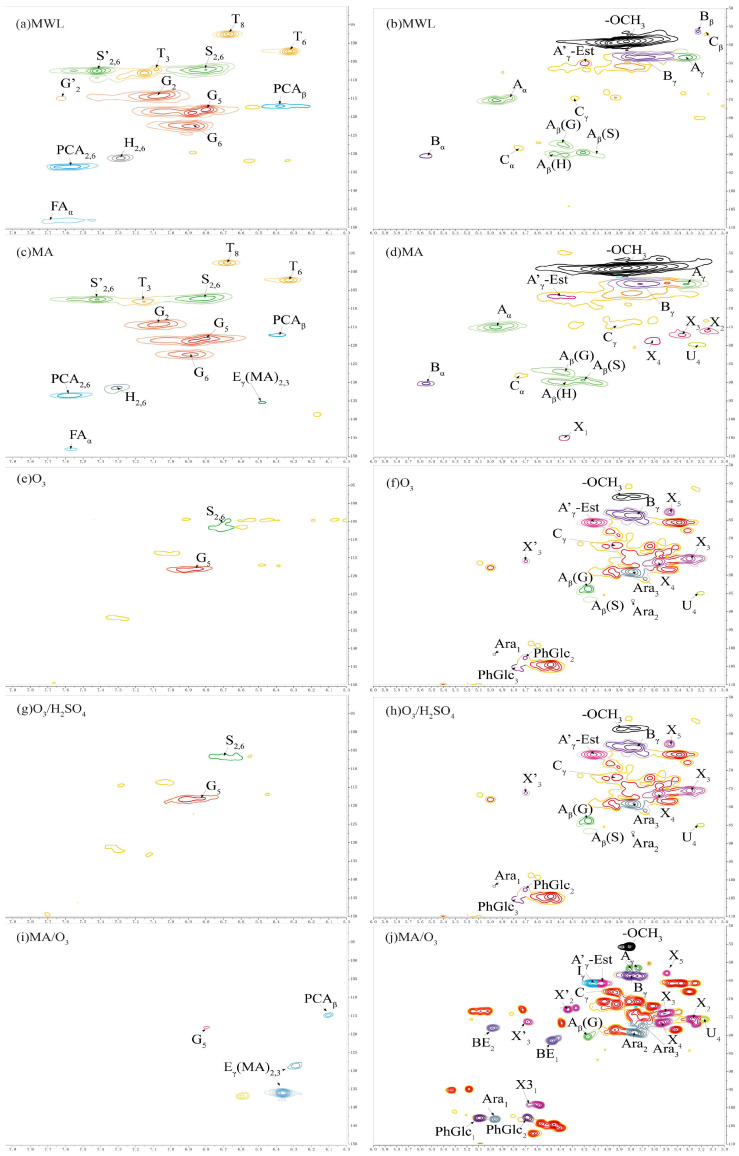
The 2DHSQCNMR of lignin (**a**,**c**,**e**,**g**,**i** are lignin phenolic ring regions; **b**,**d**,**f**,**h**,**j** are lignin side chain regions).

Previous studies were used to assign the signals of lignin and carbohydrate linkage bonds [48,49,50]. The 2D-HSQC-NMR profiles of the lignin phenolic ring region and side chain region are shown in Figure 6 and the semi-quantitative analysis of the units in the lignin structure is shown in Table 3.

#### 2.6.1. Lignin Cross-Signals

Compared with MWL, both G and S unit signals were detected in the phenolic ring region (δC/δH 90–150/8.0–6.0 ppm) of the 2D-HSQC-NMR spectra of the DL obtained under O_3_ and O_3_/H_2_SO_4_ conditions. These signals primarily included the C_5_-H_5_ signals (G5) of the guaiacyl unit and the etherified syringyl units’ C_2_-H_2_ and C_6_-H_6_ signals (S_2,6_). The S/G ratio of MWL was 0.87, while the S/G ratios of DL obtained under O_3_ and O_3_/H_2_SO_4_ conditions were 0.61 and 0.45, respectively, indicating a significant decrease in the proportion of S units. Only G-unit signals, mainly C_5_-H_5_ signals of the guaiacyl unit (G_5_), could be detected in the phenolic ring region of the lignin under MA/O_3_ conditions. In contrast, the DL obtained by MA treatment was rich in G, S, and H unit signals, which was similar to MWL. The S/G ratio in DL directly decreased from 0.81 to 0 in the MA/O_3_ condition compared to the MA condition. The reason for this was that under the strong oxidizing effect of O_3_, the aromatic ring structure in lignin underwent a 1,3-even ring addition reaction, and the phenolic ring was cleaved to generate structures such as lactone bonds. Under the further action of O_3_, the lactone bond broke and substances such as muconic acid were generated [30,51], resulting in the missing phenolic ring signal of the lignin. In contrast, lignin obtained in different separation environments was condensed to varying degrees, and the phenolic ring was essentially not ring-opened [29]. The signals at δC/δH 136.13/6.37 ppm and δC/δH 128.73/6.29 ppm for lignin obtained under MA conditions and MA/O_3_ conditions were due to the esterification of the γ-OH of lignin by MA to form E_γ_(MA)_2,3_ [28]. The signal located at δC/δH 114.81/6.11 ppm (PCA_β_) belonged to C_8_-H_8_ in p-coumaric acid. In contrast to the MWL and DL obtained from MA conditions, the DL obtained with the participation of O_3_ in the reaction did not find the signals of tricin located at δC/δH 94.2/6.60 ppm (T_8_), δC/δH 98.8/6.31 ppm (T_6_), and δC/δH 104.1/7.07 ppm (T_3_).

In the side chain region of 2D-HSQC-NMR (δC/δH 50–110/3.1–5.5 ppm), the methoxy signal was located at δC/δH 55.74/3.81 ppm (-OCH_3_). The correlation signals of C_α_-H_α_ and C_γ_-H_γ_ in the β-O-4 structure were identified at δC/δH 72.89/4.73 ppm (A_α_) and δC/δH 61.47/3.74 ppm (A_γ_), respectively. C_β_-H_β_ cross-peaks centered at δC/δH 89.31/4.09 ppm (A_β_(S)), 87.15/4.39 ppm (A_β_(G)), and 89.96/4.41 ppm (A_β_(H)) were from β-O-4 substructures linked to S, G, and H units, respectively. The C_γ_-H_γ_ signal in the resinol structure and the C_γ_-C_γ_ cross-signal at the cinnamyl alcohol end-group were located at δC/δH 67.87/3.97 ppm (C_γ_) and δC/δH 65.56/4.13 ppm (I_γ_), respectively.

The resinol structure and the phenyl coumarin structure represent the common β-β and β-5 condensation bonds in lignin, respectively. The C_α_-H_α_ signal (C_α_) was located at δC/δH 88.05/4.75 ppm in the resinol structure and the C_α_-H_α_ signal (B_α_) was located at δC/δH 90.3/5.55 ppm in the phenyl coumarin structure. However, the above signals were not found in the DL structure obtained with O_3_ participation in the reaction. As shown in Table 3, the contents of β-β bonds and β-5 bonds in MA lignin were 10% and 7%, respectively, which were increased by 9 and 5 percentage points compared to MWL. This indicated that DL underwent significant condensation in the presence of MA. However, after the introduction of O_3_, the condensation bonds of lignin were significantly reduced, with the value directly decreased from 10% to 0, which effectively inhibited the condensation of lignin.

#### 2.6.2. Carbohydrate Cross-Signals

In Figure 6, the cross-signal peaks of C_2_-H_2_, C_3_-H_3_, C_4_-H_4_, and C_5_-H_5_ of β-D-xylopyranose were located at δC/δH 75.59/3.27 ppm (X_2_), δC/δH 73.87/3.50 ppm (X_3_), δC/δH 76.29/3.55 ppm (X_4_), and δC/δH 62.86/3.49 ppm (X_5_) [52]. The cross-signal peaks of C_1_-H_1_, C_2_-H_2_, and C_3_-H_3_ of furan-type arabinose were located at δC/δH 103.11/4.97 ppm (Ara_1_), δC/δH 79.07/3.77 ppm (Ara_2_), and at δC/δH 77.38/3.71 ppm (Ara_3_) [29]. C_3_-H_3_ signals in 1-O-acetyl-β-D-xylose were located at δC/δH 99.11/4.67 ppm (X3_1_). The cross-signal peaks of C_2_-H_2_ and C_3_-H_3_ of O-acetyl-β-D-xylopyranose were located at δC/δH 72.78/4.34 ppm (X’_2_) and δC/δH 76.08/4.68 ppm (X’_3_). The C_4_-H_4_ cross-signal in 4-O-methyl-α-D-glucuronic acid was located at δC/δH 75.48/3.18 ppm (U_4_) [53].

The lignin obtained by different separation methods except MWL had different degrees of arabinose and xylose signals. This indicated that the DLs contained some hemicellulose presence, which corresponded to the results of the FT-IR. Compared with other lignin, the side chain signals of DL obtained by MA/O_3_ conditions were more complex and contained more sugar group signals, corresponding to higher glucan and xylan solubilization rates.

## 3. Materials and Methods

### 3.1. Materials

Air-dried wheat straw was harvested from Jurong (Jurong, China). It was processed to obtain stems (without internodes), ground using a Wiley mill, and screened to retain the part of 40–80 mesh. The moisture content of the wheat straw was determined to be 8.57 ± 0.1% and the wheat straw was vacuum-dried and prepared for use. Extracts from the ground wheat straw were removed using benzene/ethanol (2:1, *v*/*v*) extraction for 8 h. The extractive-free ground wheat straw was ball-milled in a PULVERISETTE 7 reinforced planetary ball-mill (Beijing Feiqi Scientific Instrument Co., Ltd., Beijing, China) at 600 rpm for 6 h at room temperature. To prevent overheating, it was set to pause for 5 min every 10 min of operation. An ozone generator (Nanjing Qiangti Drying Equipment Co., Ltd., Nanjing, China) with a flow rate of 2 g/h was used. Maleic acid (MA) was purchased from Shanghai Macklin Biochemical Technology Co., Ltd., Shanghai, China.

### 3.2. Pretreatment Procedure with MA/O_3_

The ball-milled wheat straw was homogeneously mixed with liquids (60 wt% MA, H_2_O, or 60% H_2_SO_4_) at a controlled concentration of 30%. The O_3_ holding time (0, 3, 6, or 9 min) and reaction temperature (50, 60, 70, or 80 °C) during the liquid pretreatments were adjusted accordingly. After that, the samples were centrifuged and washed to neutral. The remaining water-insoluble solids (WISs) were recovered by vacuum freeze-drying. DL was obtained by dialysis of the supernatant, rotary evaporation, and lyophilization.

### 3.3. Chemical Composition

The chemical compositions of the wheat straw raw material and final products (WISs) were determined according to the method of the U.S. Department of Energy NREL/TP-510-42618 [54]. The amount of acid-soluble lignin was measured by its absorption at 205 nm using a UV spectrophotometer (TU-1900, Beijing General Instrument Co., Beijing, China). The glycosyl content was detected and analyzed using an HPLC system (Agilent 1200 Series, Santa Clara, CA, USA) equipped with a BioRad Aminex HPX-87H column (300 mm × 7.8 mm) and a refractive index detector (RID) (RI-101, Shodex, Tokyo, Japan). The contents of lignin, glucan, xylan, and extractives in the ground wheat straw were obtained as 21.95 ± 0.2%, 36.8 ± 0.3%, 20.9 ± 0.2%, and 3.49 ± 0.1%, respectively.

### 3.4. Analytical Procedures

Crystallinity was determined using a combined multifunctional X-ray diffractometer (XRD Ultima IV, Tokyo, Japan) for the ground wheat straw and the 6 h ball-milled wheat straw. The 2θ angles ranged from 10° to 40°.

The particle size and zeta potential were determined by dynamic light scattering (DLS). Here, 10 mg of samples was dispersed in 150 mL of deionized water and sonicated for 30 min until completely dissolved. Then, an appropriate amount of this solution was taken to measure the particle size and zeta potential of the lignin by DLS.

Conductivity titration was used to determine the carboxyl group content in lignin [55].

### 3.5. Lignin Structure Analysis

MWL was isolated based on previous studies [28]. A total of 6–8 mg of the sample was weighed in a crucible and its thermal stability was measured using a thermogravimetric analyzer (TG) in a N_2_ environment. The temperature range was set between 30 and 800 °C, with a gas flow rate of 20 mL/min and a heating rate of 10 K/min.

UV spectra were obtained with a UV spectrophotometer (TU-1900, Beijing General Instrument, Beijing, China) in the wavelength range of 200–600 nm. The FTIR spectra of the lignin were measured by an FT-IR spectrometer (VERTEX 80V, Bruker, Berlin, Germany). The 2D-HSQC-NMR of lignin was conducted using Bruker AVANCE 600 MHz spectrometer, following our previous work [56]. The 2D-HSQC-NMR spectra obtained were analyzed using MestRenova-14.0.0-23239 software. The semi-quantitative calculation of lignin structural units was based on existing references [57,58].

Gel permeation chromatography analysis (GPC, LC-20A, Shimadzu Co., Kyoto, Japan) was used to determine the molecular weight of the lignin to obtain the weight-average molecular weight (M_W_), number-average molecular weight (M_n_), and polydispersity index (PI, M_W_/M_n_). The acetylated lignin was dissolved in THF at a 1 mg/mL concentration [28]. The gel column, which was 300 mm × 8.0 mm in size, was calibrated using polystyrene standards with peak average molecular weights of 43,600, 30,000, 20,000, 10,000, 4050, and 2400 Da. The elution rate of THF was maintained at 1 mL/min, while the column temperature was set at 40 °C.

## 4. Conclusions

In this study, a novel biomass refining method was developed under the following experimental conditions: ball-milling for 6 h, MA concentration of 60%, wheat straw concentration of 30%, and O_3_ holding time of 9 min. Under these conditions, the lignin, dextran, and xylan removal ratios from the wheat straw were 38.07 ± 0.2%, 71.98 ± 0.1%, and 31.44 ± 0.1%, respectively. The results demonstrated the feasibility of using the MA/O_3_ system to separate components of lignocellulosic raw materials. The lignin obtained from this separation process introduced a substantial number of carboxyl groups through the esterification by MA and oxidation by O_3_, with a carboxyl group content of approximately 2.96 mmol/g. Due to the presence of O_3_, the lignin phenolic ring opened and the side chains were oxidized, preventing the condensation of G and S units. As a result, the β-β and β-5 condensation bonds almost disappeared. The lignin obtained in this study had a small average molecular weight of 7758 and a particle size of only 221.6 nm. This nanoscale lignin has the potential to be used in functional materials. However, this study is only a theoretical study of lignin and did not perform effective application research, which needs to be further explored.

## Figures and Tables

**Table 1:** WIS ^a^ yield and component removal at a reaction temperature of 60 °C and O_3_ holding time of 9 min.

Item	Temp. ^c^ (°C)	O_3_ Holding Time (min)	Ground Wheat Straw				Ball-Milled Wheat Straw			
			MA ^d^	MA/O_3_	O_3_^e^	O_3_/H_2_SO_4_^f^	MA	MA/O_3_	O_3_	O_3_/H_2_SO_4_
Yield (%)	60 °C	0	78.40 ± 0.3	N/A			63.66 ± 0.5	N/A		
		9	N/A ^g^	81.20 ± 0.4	92.33 ± 0.5	94.33 ± 0.3	N/A	63.05 ± 0.3	82.67 ± 0.4	70.33 ± 0.6
Lignin removal ratio ^b^ (%)		0	18.80 ± 0.2	N/A			28.46 ± 0.8	N/A		
		9	N/A	23.32 ± 0.1	4.77 ± 0.7	14.32 ± 0.3	N/A	38.07 ± 0.2	15.78 ± 0.7	28.25 ± 0.7
Dextran removal ratio ^b^ (%)		0	13.77 ± 0.3	N/A			22.28 ± 0.3	N/A		
		9	N/A	21.53 ± 0.3	5.15 ± 0.6	10.54 ± 0.2	N/A	31.44 ± 0.1	14.95 ± 0.6	25.98 ± 0.9
Xylan removal ratio ^b^ (%)		0	13.76 ± 0.4	N/A			55.16 ± 0.4	N/A		
		9	N/A	25.35 ± 0.2	10.15 ± 0.4	17.53 ± 0.7	N/A	71.98 ± 0.1	23.37 ± 0.3	59.34 ± 0.5

^a^ Water–insoluble solids. ^b^ The removal ratio of components was calculated based on the original content of each component in the ground wheat straw. ^c^ Temperature. ^d^ Maleic acid. ^e^ Ozone. ^f^ Sulfuric acid. ^g^ Not applicable.

**Table 2:** Weight-average (M_w_), number-average (M_n_) molecular weights, and polydispersity indexes (PI, M_w_/M_n_) of lignin.

Name of Sample	MWL	MA	O_3_	MA/O_3_	O_3_/H_2_SO_4_
M_w_	14,678	8105	9730	7758	7990
M_n_	4258	3563	1832	1954	1802
PI	3.45	2.27	5.31	3.97	4.43

**Table 3:** HSQC semi–quantitative analysis of lignin structural units.

Characteristics	MWL	MA	O_3_	O_3_/H_2_SO_4_	MA/O_3_
Lignin interunit linkages					
β-O-4	50	52	0	0	0
β-β	1	10	0	0	0
β-5	2	7	0	0	0
Condensed degree ^a^	6	10	0	0	0
Lignin aromatic units					
G	51	53	62	69	100
S	45	43	38	31	0
H	4	4	0	0	0
S/G	0.87	0.81	0.61	0.45	0

^a^ Condensed degree, % = 100 × (IB_α_ + IC_α_)/(IA_α_ + IB_α_ + IC_α_), which refers to the integral value of each signal in 2D–HSQC–NMR.

## Data Availability

The data presented in this study are available upon request from the corresponding author.

## Supplementary Materials

The following supporting information can be downloaded at: https://www.mdpi.com/article/10.3390/molecules29133228/s1, Figure S1: Reactions between O_3_ and lignin; Table S1: WIS yield and the removal ratio of each component; Table S2: T_m_ and residual carbon of lignin.

## References

[1] Jafari-Petroudy S.R., Resalati H., Rezayati-Charani P. (2011). Newsprint from Soda Bagasse Pulp in Admixture with Hardwood CMP Pulp. Bioresources.

[2] Sarwar M., Khan M.A., Mahr-un N. (2003). Nitrogen retention and chemical composition of urea treated wheat straw ensiled with organic acids or fermentable carbohydrates. Asian-Australas. J. Anim. Sci..

[3] Taranenko A., Kulyk M., Galytska M., Taranenko S., Rozhko I. (2021). Dynamics of soil organic matter in Panicum virgatum sole crops and intercrops. Zemdirb.-Agric..

[4] Brummer V., Jurena T., Hlavacek V., Omelkova J., Bebar L., Gabriel P., Stehlik P. (2014). Enzymatic hydrolysis of pretreated waste paper—Source of raw material for production of liquid biofuels. Bioresour. Technol..

[5] Cekmecelioglu D., Uncu O.N. (2013). Kinetic modeling of enzymatic hydrolysis of pretreated kitchen wastes for enhancing bioethanol production. Waste Manag..

[6] Lai C.H., Yang C.D., Jia Y., Xu X., Wang K., Yong Q. (2022). Lignin fractionation to realize the comprehensive elucidation of structure-inhibition relationship of lignins in enzymatic hydrolysis. Bioresour. Technol..

[7] Li K.Y., Zhong W., Li P.H., Ren J.P., Jiang K.J., Wu W.J. (2023). Antibacterial mechanism of lignin and lignin-based antimicrobial materials in different fields. Int. J. Biol. Macromol..

[8] Ma Y.L., Dai J.X., Wu L.L., Fang G.Z., Guo Z.H. (2017). Enhanced anti-ultraviolet, anti-fouling and anti-bacterial polyelectrolyte membrane of polystyrene grafted with trimethyl quaternary ammonium salt modified lignin. Polymer.

[9] Figueiredo P., Lintinen K., Kiriazis A., Hynninen V., Liu Z.H., Bauleth-Ramos T., Rahikkala A., Correia A., Kohout T., Sarmento B. (2017). In vitro evaluation of biodegradable lignin-based nanoparticles for drug delivery and enhanced antiproliferation effect in cancer cells. Biomaterials.

[10] Frangville C., Rutkevicius M., Richter A.P., Velev O.D., Stoyanov S.D., Paunov V.N. (2012). Fabrication of Environmentally Biodegradable Lignin Nanoparticles. Chemphyschem.

[11] de Albuquerque T.L., Cavalcante V.G.C., Rocha W.D., de Macedo A.C., Rocha M.V.P. (2024). Hydrogels based on lignin extracted from cashew apple bagasse and its application in antimicrobial wound dressings. Int. J. Biol. Macromol..

[12] Li J., Xu X., Ma X., Cui M., Wang X., Chen J., Zhu J., Chen J. (2024). Antimicrobial Nonisocyanate Polyurethane Foam Derived from Lignin for Wound Healing. ACS Appl. Bio Mater..

[13] Domínguez-Robles J., Cárcamo-Martínez A., Stewart S.A., Donnelly R.F., Larrañeta E., Borrega M. (2020). Lignin for pharmaceutical and biomedical applications—Could this become a reality?. Sustain. Chem. Pharm..

[14] Norouzi M., Rafienia M., Hosseini S. (2023). Characterization and biological evaluation of new PLGA/fibrin/lignin biocomposite electrospun scaffolds. Phys. Scr..

[15] Kandil H., Ekram B., Abo-Zeid M.A.M. (2023). Cytocompatibility of MG-63 osteosarcoma cells on chitosan/hydroxyapatite/lignin hybrid composite scaffold in vitro. Biomed. Mater..

[16] Winters C., Carsi M., Sanchis M.J., Culebras M., Collins M.N. (2024). On the design of lignin reinforced acrylic acid/hyaluronic acid adhesive hydrogels with conductive PEDOT:HA nanoparticles. Int. J. Biol. Macromol..

[17] Alaoui C.H., Rethoré G., Weiss P., Fatimi A. (2023). Sustainable Biomass Lignin-Based Hydrogels: A Review on Properties, Formulation, and Biomedical Applications. Int. J. Mol. Sci..

[18] Garlapati V.K., Chandel A.K., Kumar S.P.J., Sharma S., Sevda S., Ingle A.P., Pant D. (2020). Circular economy aspects of lignin: Towards a lignocellulose biorefinery. Renew. Sustain. Energy Rev..

[19] Sun Q., Chen W.J., Pang B., Sun Z.H., Lam S.S., Sonne C., Yuan Q. (2021). Ultrastructural change in lignocellulosic biomass during hydrothermal pretreatment. Bioresour. Technol..

[20] Hsu T.C., Guo G.L., Chen W.H., Hwang W.S. (2010). Effect of dilute acid pretreatment of rice straw on structural properties and enzymatic hydrolysis. Bioresour. Technol..

[21] Chen J.H., Xu J.K., Huang P.L., Sun R.C. (2016). Effect of alkaline pretreatment on the preparation of regenerated lignocellulose fibers from bamboo stem. Cellulose.

[22] Zakaria S.M., Idris A., Alias Y. (2017). Lignin Extraction from Coconut Shell Using Aprotic Ionic Liquids. Bioresources.

[23] Zhang X.M., Yuan T.Q., Peng F., Xu F., Sun R.C. (2010). Separation and Structural Characterization of Lignin from Hybrid Poplar Based on Complete Dissolution in DMSO/LiCl. Sep. Sci. Technol..

[24] Rinaldi R., Jastrzebski R., Clough M.T., Ralph J., Kennema M., Bruijnincx P.C.A., Weckhuysen B.M. (2016). Paving the Way for Lignin Valorisation: Recent Advances in Bioengineering, Biorefining and Catalysis. Angew. Chem.-Int. Ed..

[25] Zhao X., Yang Y.Y., Xu J.Y., Guo Y.Z., Zhou J.H., Wang X. (2022). Ni_12_P_5_/P-N-C Derived from Natural Single-Celled Chlorella for Catalytic Depolymerization of Lignin into Monophenols. ACS Omega.

[26] Zhu J.J., Chen L.H., Gleisner R., Zhu J.Y. (2019). Co-production of bioethanol and furfural from poplar wood via low temperature (≤90 °C) acid hydrotropic fractionation (AHF). Fuel.

[27] Ma Q.L., Zhu J.J., Gleisner R., Yang R.D., Zhu J.Y. (2018). Valorization of Wheat Straw Using a Recyclable Hydrotrope at Low Temperatures (≤90 °C). ACS Sustain. Chem. Eng..

[28] Cai C., Hirth K., Gleisner R., Lou H.M., Qiu X.Q., Zhu J.Y. (2020). Maleic acid as a dicarboxylic acid hydrotrope for sustainable fractionation of wood at atmospheric pressure and ≤100 °C: Mode and utility of lignin esterification. Green Chem..

[29] Su C., Hirth K., Liu Z.L., Cao Y.F., Zhu J.Y. (2021). Maleic acid hydrotropic fractionation of wheat straw to facilitate value-added multi-product biorefinery at atmospheric pressure. Glob. Chang. Biol. Bioenergy.

[30] Ma R.S., Xu Y., Zhang X. (2015). Catalytic Oxidation of Biorefinery Lignin to Value-added Chemicals to Support Sustainable Biofuel Production. Chemsuschem.

[31] Mamleeva N.A., Autlov S.A., Fionov A.V., Bazarnova N.G., Lunin V.V. (2009). The oxidative destruction of lignin in the ozonation of wood. Russ. J. Phys. Chem. A.

[32] Shi C., Zhang S., Wang W., Linhardt R.J., Ragauskas A.J. (2020). Preparation of Highly Reactive Lignin by Ozone Oxidation: Application as Surfactants with Antioxidant and Anti-UV Properties. ACS Sustain. Chem. Eng..

[33] Khadre M.A., Yousef A.E., Kim J.G. (2001). Microbiological aspects of ozone applications in food: A review. J. Food Sci..

[34] Seta F.T., An X.Y., Liu L.Q., Zhang H., Yang J., Zhang W., Nie S.X., Yao S.Q., Cao H.B., Xu Q.L. (2020). Preparation and characterization of high yield cellulose nanocrystals (CNC) derived from ball mill pretreatment and maleic acid hydrolysis. Carbohydr. Polym..

[35] Tripathi S.K., Bhardwaj N.K., Ghatak H.R. (2020). Developments in Ozone-Based Bleaching of Pulps. Ozone-Sci. Eng..

[36] Garcia J.C., Lopez F., Perez A., Pelach M.A., Mutje P., Colodette J.L. (2010). Initiating ECF bleaching sequences of eucalyptus kraft pulps with Z/D and Z/E stages. Holzforschung.

[37] Travaini R., Martín-Juarez J., Lorenzo-Hernando A., Bolado-Rodríguez S. (2016). Ozonolysis: An advantageous pretreatment for lignocellulosic biomass revisited. Bioresour. Technol..

[38] Wang H.Y., Zhao L.H., Ren J.L., He B.H. (2022). Structural Changes of Alkali Lignin under Ozone Treatment and Effect of Ozone-Oxidized Alkali Lignin on Cellulose Digestibility. Processes.

[39] Liu Z.L., Meng L.K., Chen J.Q., Cao Y.F., Wang Z.G., Ren H. (2016). The utilization of soybean straw III: Isolation and characterization of lignin from soybean straw. Biomass Bioenergy.

[40] Delmas G.H., Benjelloun-Mlayah B., Le Bigot Y., Delmas M. (2011). Functionality of Wheat Straw Lignin Extracted in Organic Acid Media. J. Appl. Polym. Sci..

[41] Mohammadpour R., Sadeghi G.M.M. (2020). Potential use of black liquor as lignin source for synthesis of polyurethane foam. J. Polym. Res..

[42] Shukry N., Fadel S.M., Agblevor F.A., Ei-Kalyoubi S.F. (2008). Some physical properties of acetosolv lignins from bagasse. J. Appl. Polym. Sci..

[43] Faravelli T., Frassoldati A., Migliavacca G., Ranzi E. (2010). Detailed kinetic modeling of the thermal degradation of lignins. Biomass Bioenergy.

[44] Yang J.Y., Yu Q.F., Li M.F. (2023). Freeze-thaw assisted maleic acid pretreatment of eucalyptus to prepare cellulose nanocrystals and degraded lignin. Bioresour. Technol..

[45] Figueirêdo M.B., Heeres H.J., Deuss P.J. (2020). Ozone mediated depolymerization and solvolysis of technical lignins under ambient conditions in ethanol. Sustain. Energy Fuels.

[46] Wang R., Chen C.L., Gratzl J.S. (2004). Ozonation of pine kraft lignin in alkaline solution. Part 1: Ozonation, characterization of kraft lignin and its ozonated preparations. Holzforschung.

[47] Xiao M.Z., Chen W.J., Hong S., Pang B., Cao X.F., Wang Y.Y., Yuan T.Q., Sun R.C. (2019). Structural characterization of lignin in heartwood, sapwood, and bark of eucalyptus. Int. J. Biol. Macromol..

[48] Qiao X.L., Zhao C., Shao Q.J., Hassan M. (2018). Structural Characterization of Corn Stover Lignin after Hydrogen Peroxide Presoaking Prior to Ammonia Fiber Expansion Pretreatment. Energy Fuels.

[49] Xie D., Gan T., Su C., Han Y., Liu Z.L., Cao Y.F. (2020). Structural characterization and antioxidant activity of water-soluble lignin-carbohydrate complexes (LCCs) isolated from wheat straw. Int. J. Biol. Macromol..

[50] Khongchamnan P., Wanmolee W., Laosiripojana N., Champreda V., Suriyachai N., Kreetachat T., Sakulthaew C., Chokejaroenrat C., Imman S. (2021). Solvothermal-Based Lignin Fractionation From Corn Stover: Process Optimization and Product Characteristics. Front. Chem..

[51] Schutyser W., Renders T., Van den Bosch S., Koelewijn S.F., Beckham G.T., Sels B.F. (2018). Chemicals from lignin: An interplay of lignocellulose fractionation, depolymerisation, and upgrading. Chem. Soc. Rev..

[52] Jiang B., Cao T.Y., Gu F., Wu W.J., Jin Y.C. (2017). Comparison of the Structural Characteristics of Cellulolytic Enzyme Lignin Preparations Isolated from Wheat Straw Stem and Leaf. ACS Sustain. Chem. Eng..

[53] Giummarella N., Lawoko M. (2016). Structural Basis for the Formation and Regulation of Lignin-Xylan Bonds in Birch. ACS Sustain. Chem. Eng..

[54] Ibanez A.B., Bauer S. (2014). Downscaled method using glass microfiber filters for the determination of Klason lignin and structural carbohydrates. Biomass Bioenergy.

[55] Li X.K., Zhao X., Zhu H.W., Wang X., Zhou J.H. (2022). Research of Inhibiting Lignin Condensation by Ethylene Glycol During DES Pretreatment Process. Trans. China Pulp Pap..

[56] Su C., Hirth K., Liu Z.L., Cao Y.F., Zhu J.Y. (2021). Acid hydrotropic fractionation of switchgrass at atmospheric pressure using maleic acid in comparison with p-TsOH: Advantages of lignin esterification. Ind. Crop. Prod..

[57] Li N., Li Y.D., Yoo C.G., Yang X.H., Lin X.L., Ralph J., Pan X.J. (2018). An uncondensed lignin depolymerized in the solid state and isolated from lignocellulosic biomass: A mechanistic study. Green Chem..

[58] Yue P.P., Hu Y.J., Fu G.Q., Sun C.X., Li M.F., Peng F., Sun R.C. (2018). Structural Differences between the Lignin-Carbohydrate Complexes (LCCs) from 2-and 24-Month-Old Bamboo (*Neosinocalamus affinis*). Int. J. Mol. Sci..

