# Supplementary material for: The Synergetic Reduction of the Condensation Degree of Dissolved Lignin (DL) during the Refining Process of Wheat Straw Biomass Based on the MA/O3 System
Source: Molecules. 2024 Jul 8;29(13):3228. doi: 10.3390/molecules29133228 (PMC11243111; doi:10.3390/molecules29133228)
Supplement: Supplementary file 1 [file molecules-29-03228-s001.zip › molecules-3059303-supplementary.pdf]

The Synergetic Reduction of the Condensation Degree of Dissolved Lignin (DL) during the Refining Process of Wheat Straw Biomass Based on the MA/O_3 System

Xiuguang Chen [1], Zhulan Liu [1,2,*], Zhenyu Zhou [2], Ren'ai Li [1], Lizi Li [2] and Yunfeng Cao [1,*]

[1] Jiangsu Co-Innovation Center for Efficient Processing and Utilization of Forest Resources, Jiangsu Provincial Key Lab Pulp & Paper Science and Technology, Nanjing Forestry University, Nanjing 210037, China; chenxiuguang@njfu.edu.cn (X.C.); lirenai@njfu.edu.cn (R.L.)

[2] Zhejiang Kan New Materials Co., Ltd., Lishui 323300, China; cxg19980906@163.com (Z.Z.); 18168034156@163.com (L.L.)

*Correspondence: liuzhulan@njfu.edu.cn (Z.L.); yunfcao@163.com (Y.C.)

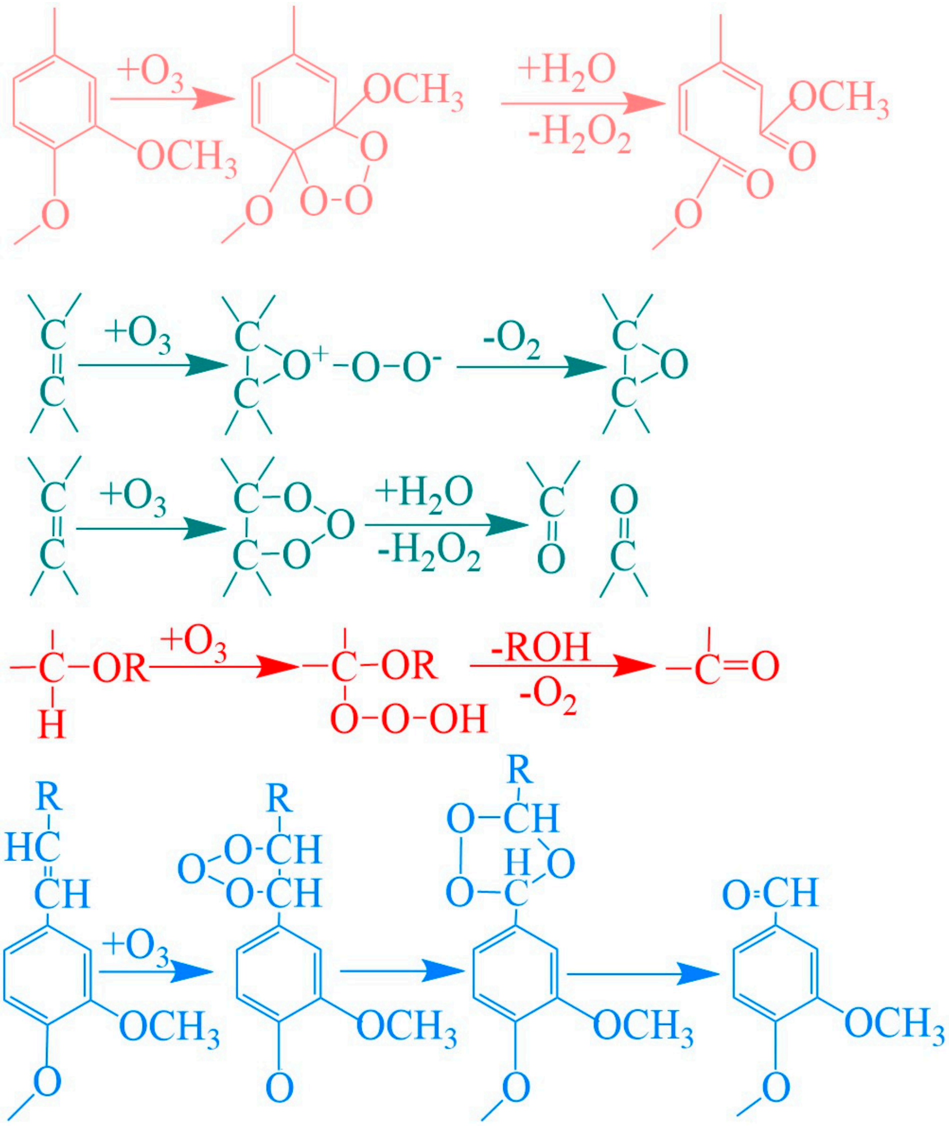

Figure S1. Reactions between O_3 and lignin

Table S1. WIS[a] yield and the removal ratio of each component

Item	Temp[c]. (℃)	O_3 holding Time (min)	Ground wheat straw				Ball-milled wheat straw			
			MA[d]	MA/O_3	O_3[e]	O_3/H_2SO_4[f]	MA	MA/O_3	O_3	O_3/H_2SO_4
Yield (%)	50	0	85.40±0.4	N/A[g]			64.00±0.5	N/A		
		3	N/A	88.60±0.3	93.67±0.3	95.67±0.4	N/A	66.00±0.5	87.67±0.6	73.00±0.6
		6		76.20±0.7	89.33±0.3	93.00±0.5		65.67±0.2	87.00±0.1	71.67±0.3
		9		80.80±0.4	91.33±0.8	93.67±0.7		64.00±0.3	84.33±0.2	71.25±0.7
	60	0	78.40±0.3	N/A			63.66±0.5	N/A		
		3	N/A	85.20±0.4	91.00±0.9	95.67±0.2	N/A	66.33±0.1	82.67±0.3	73.00±0.1
		6		80.00±0.6	90.66±0.7	93.37±0.5		65.67±0.8	83.33±0.9	72.33±0.5
		9		81.20±0.4	92.33±0.5	94.33±0.3		63.05±0.3	82.67±0.4	70.33±0.6
	70	0	79.80±0.6	N/A			64.33±0.8	N/A		
		3	N/A	83.40±0.5	94.66±0.6	95.00±0.2	N/A	66.66±0.3	80.33±1.0	75.67±0.5
		6		81.40±0.4	90.66±0.9	95.33±0.1		64.67±0.6	78.33±0.8	72.35±0.3
		9		83.60±0.2	91.00±0.5	94.21±0.4		64.33±0.9	77.87±0.4	73.00±0.3
	80	0	77.20±0.7	N/A			63.00±0.4	N/A		
		3	N/A	77.00±0.9	92.66±0.8	95.76±0.7	N/A	69.33±0.5	96.83±0.7	76.00±0.9
		6		76.80±0.7	86.00±0.8	93.47±0.6		68.00±0.4	95.67±0.3	75.33±0.2
		9		79.14±0.1	87.00±0.3	92.37±0.3		70.33±0.6	90.33±0.8	74.67±0.9
Lignin removal ratio[b] (%)	50	0	16.58±0.4	N/A			25.31±0.3	N/A		
		3	N/A	17.80±0.5	3.24±0.2	10.45±0.9	N/A	30.62±0.6	9.90±0.7	20.86±0.9
		6		20.93±0.6	3.97±0.7	14.12±0.8		30.78±0.5	10.54±0.4	21.66±0.3
		9		18.00	3.46	12.32±0.5		32.01±	11.96±	23.85

				±0.8	±0.7			0.4	0.1	±0.2
	60	0	18.80±0.2	N/A			28.46±0.8	N/A		
		3	N/A	19.96±0.9	4.74±0.3	11.34±0.4	N/A	31.23±0.6	14.04±0.1	21.95±0.2
		6		25.21±1	5.74±0.7	16.11±0.6		31.62±0.8	15.31±0.3	25.95±0.4
		9		23.32±0.1	4.77±0.7	14.32±0.3		38.07±0.2	15.78±0.7	28.25±0.7
	70	0	20.14±0.5	N/A			29.50±0.1	N/A		
		3	N/A	21.86±0.8	2.00±0.2	9.78±0.8	N/A	31.76±0.9	7.93±0.6	16.56±0.9
		6		26.54±0.9	2.19±0.4	10.99±0.5		33.07±0.5	9.21±0.4	17.98±0.7
		9		24.35±0.7	2.13±0.6	8.7±0.2		35.53±0.7	9.45±0.2	18.97±0.2
	80	0	23.75±0.6	N/A			30.53±0.9	N/A		
		3	N/A	24.48±1	1.08±0.5	8.95±0.4	N/A	32.38±0.2	4.85±0.6	14.11±0.5
		6		26.70±0.3	1.91±0.9	9.22±0.6		34.82±0.9	6.40±0.7	17.27±0.4
		9		25.88±0.4	1.68±0.8	8.52±0.8		34.98±0.6	6.91±0.2	17.68±0.2
Dextran removal ratio[b] (%)	50	0	11.10±0.3	N/A			19.65±0.9	N/A		
		3	N/A	11.56±0.5	2.33±0.4	9.11±0.1	N/A	19.80±0.8	11.84±0.4	17.34±0.7
		6		13.96±0.7	3.25±0.2	10.93±0.2		20.58±0.5	12.33±0.6	18.38±0.9
		9		11.72±0.6	2.41±0.7	10.19±0.3		20.64±0.1	12.56±0.4	19.25±0.1
	60	0	13.77±0.3	N/A			22.88±0.3	N/A		
		3	N/A	14.07±0.4	4.83±0.7	10.16±0.5	N/A	23.76±0.1	13.20±0.4	19.14±0.7
		6		24.78±0.8	5.96±0.2	12.26±0.9		26.25±0.3	14.85±0.4	23.47±0.6
		9		21.53±0.3	5.15±0.6	10.54±0.2		31.44±0.1	14.95±0.6	25.98±0.9
	70	0	15.84±1	N/A			23.03±0.3	N/A		

		3		17.71±0.2	0.98±0.8	9.28±0.4		25.86±0.2	9.85±0.5	19.04±0.9
		6	N/A	25.51±0.6	1.59±0.3	9.71±0.1	N/A	27.97±0.8	11.06±0.7	22.46±0.3
		9		23.22±0.9	1.15±0.1	8.39±0.2		28.40±0.9	11.51±0.8	23.30±0.2
	80	0	20.06±0.1	N/A			26.05±0.3	N/A		
		3		23.58±0.8	0.25±0.7	8.55±0.4		26.47±0.2	6.24±0.7	12.83±0.4
		6	N/A	27.69±0.8	0.74±0.6	9.45±0.2	N/A	28.86±0.5	8.73±0.1	13.61±0.2
		9		25.42±0.1	0.66±0.3	7.60±0.2		29.25±0.9	9.11±0.8	15.96±0.7
Xylan removal ratio[b] (%)	50	0	10.29±0.3	N/A			53.58±0.5	N/A		
		3		11.60±0.3	5.41±0.7	10.42±0.6		55.79±0.4	18.43±0.8	52.89±0.9
		6	N/A	25.68±0.1	7.11±0.6	18.46±0.8	N/A	58.61±0.3	19.56±0.5	54.62±0.8
		9		20.07±0.2	5.91±0.9	17.19±0.7		62.05±0.2	20.35±0.7	58.29±0.2
	60	0	13.76±0.4	N/A			55.16±0.4	N/A		
		3		15.61±0.5	9.11±0.3	16.51±0.4		57.02±0.8	21.26±0.9	54.36±0.1
		6	N/A	26.28±0.5	10.71±0.1	18.73±0.2	N/A	59.49±0.2	22.08±0.7	56.80±1
		9		25.35±0.2	10.15±0.4	17.53±0.7		71.98±0.1	23.37±0.3	59.34±0.5
	70	0	16.12±0.5	N/A			56.61±0.8	N/A		
		3		17.01±0.3	3.98±0.8	16.31±0.1		57.35±0.9	13.74±0.5	52.73±0.3
		6	N/A	30.02±0.7	4.44±0.5	17.07±0.3	N/A	62.61±0.6	16.20±0.4	53.93±0.2
		9		26.19±0.6	4.31±0.2	15.12±0.8		65.22±0.8	16.51±0.6	54.14±0.1
	80	0	26.94±0.2	N/A			58.16±0.6	N/A		
		3	N/A	29.58±0.2	2.63±0.4	9.45±0.9	N/A	65.43±0.1	11.08±0.6	48.61±0.8
		6		31.91±0.1	3.85±0.5	10.02±1		66.42±0.2	12.90±0.3	52.08±0.7

9	30.17 ±0.6	3.22 ±0.8	8.06±0.8	67.22± 0.4	13.08± 0.9	53.80 ±0.2

[a] Water-insoluble solids. [b]: The removal ratio of components is calculated based on the original content of each component in the ground wheat straw. [c] Temperature. [d] Maleic acid. [e] Ozone. [f] Sulfuric acid. [g] Not applicable.

Table S2. T_m and residual carbon of lignin

Name of sample	MWL	MA	O_3	MA/O_3	O_3/H_2SO_4
T_m(℃)	374	337	262	256	265
residual carbon （%）	25.91	34.98	27.73	22.02	29.94
